# Response of Controlled Cell Load Biofilms to Cold Atmospheric Plasma Jet: Evidence of Extracellular Matrix Contribution

**DOI:** 10.3390/life11070694

**Published:** 2021-07-15

**Authors:** Maritxu Labadie, Frédéric Marchal, Nofel Merbahi, Elisabeth Girbal-Neuhauser, Catherine Fontagné-Faucher, Claire-Emmanuelle Marcato-Romain

**Affiliations:** 1 UPS, IUT “A”, LBAE EA 4565 (Laboratoire de Biotechnologies Agroalimentaire et Environnementale), Université de Toulouse, IUT Site d’AUCH, 24 rue d’Embaquès, F-32000 Auch, France; labadie.maritxu@hotmail.fr (M.L.); elisabeth.neuhauser@iut-tlse3.fr (E.G.-N.); cathy.faucher@iut-tlse3.fr (C.F.-F.); 2 UPS, INPT, CNRS, LAPLACE UMR 5213 (Laboratoire Plasma et Conversion d’Energie), Université de Toulouse, 118 Route de Narbonne, F-31062 Toulouse, France; frederic.marchal@laplace.univ-tlse.fr (F.M.); nofel.merbahi@laplace.univ-tlse.fr (N.M.)

**Keywords:** *P. aeruginosa*, *L. citreum*, bacterial survival, extracellular polymeric substances, ATR-FTIR, non-thermal plasma, biofilm

## Abstract

Aim: Study of the biocidal effect of a cold atmospheric-pressure plasma in ambient air on single-species bacterial biofilms with controlled cell density, characterized by different extracellular matrices. Methods and results: Two bacterial strains were chosen to present different Gram properties and contrasted extracellular matrices: *Pseudomonas aeruginosa* ATCC 15442 (Gram-negative), and *Leuconostoc citreum* NRRL B-1299 (Gram-positive). *P. aeruginosa* biofilm exhibits a complex matrix, rich in proteins while *L. citreum* presents the specificity to produce glucan-type exopolysaccharides when grown in the presence of sucrose. Plasma was applied on both surface-spread cells and 24-h grown biofilms with controlled cell loads over 5, 10, or 20 min. Surface-spread bacteria showed a time dependent response, with a maximal bacterial reduction of 2.5 log after 20 min of treatment. On the other hand, in our experimental conditions, no bactericidal effect could be observed when treating biofilms of *P. aeruginosa* and glucan-rich *L. citreum*. Conclusions: For biofilms presenting equivalent cell loads, the response to plasma treatment seemed to depend on the properties of the extracellular matrix characterized by infrared spectroscopy, scanning electron microscopy, or dry weight. Significance and impact of study: Both cell load standardization and biofilm characterization are paramount factors to consider the biocide effect of plasma treatments. The extracellular matrix could affect the plasma efficacy by physical and/or chemical protective effects.

## 1. Introduction

Biofilms are microbial communities attached to a surface and embedded in a self-produced matrix of extracellular polymeric substances. Biofilms can colonize biotic or abiotic surfaces, hence leading to various detrimental effects in the medical, food, and industrial sectors [1]. The extracellular matrix (ECM) consists of a fully hydrated cross-linked complex mixture of proteins, polysaccharides, and extracellular DNA (eDNA). The biofilm matrix has diverse functions, such as maintaining the structural integrity of the biofilm and protecting cells from adverse environmental conditions [2]. Moreover, biofilm cells are in a different physiologic state compared to planktonic cells, and it is well known that bacteria growing within a biofilm structure have a higher resistance to desiccation and antimicrobials, compared to their planktonic counterparts. This enhanced tolerance has been attributed to a slower metabolism, protective proteins, and the presence of the matrix itself which can provide a physical barrier to prevent the penetration of antimicrobials [1,3].

Cold atmospheric-pressure plasma (CAP), is considered as an innovative biofilm control strategy, and is currently developing as an environmentally-friendly method in order to reduce bacterial biofilms on surfaces [4]. Plasma, considered as the fourth state of matter, is defined as a highly reactive ionized gas with a net neutral charge. CAP can be experimentally generated by devices using an electromagnetic field and different noble gases, or even atmospheric air [5,6]. A huge variety of different plasma sources has been developed, the majority of which are based either on a dielectric barrier discharge (DBD), corona, or gliding arc discharges. Although the exact mechanism of CAP bactericidal action has still to be elucidated, it is nevertheless known that atmospheric air CAP is a source of multiple reactive species, including reactive oxygen and nitrogen species (RONS), excited molecules, and UV photons that contribute to the antimicrobial properties of plasmas [7,8,9]. Due to its decontaminating properties, CAP technology has been adopted for a wide range of applications going from food safety to biomedical domains, including oral health, wound healing, sterilization, and decontamination of inorganic and biomaterials [10,11]. The bactericidal effect of CAP on planktonic bacteria has been widely investigated [8,12], while only fewer reports are available for biofilm inactivation. CAP efficiency against biofilm-forming bacteria was found less efficacious compared to planktonic bacteria and the duration of the treatment was usually increased [13,14,15,16]. However, numerous factors could impact the comparison of inactivation rates and/or efficacy of CAP treatments between studies and should also be considered in order to have a better understanding of the involved mechanisms: (i) Plasma-related factors, such as gas discharge composition, voltage and frequency of power supply, distance between biofilm surface and plasma electrode, and exposure time; and (ii) microbial-related factors, including bacterial concentration, biofilm thickness, and the nature and structure of extracellular polymers [12] . For instance, several reports underlined a reduced plasma efficacy when bacterial concentrations increased, as for planktonic *E. coli* [17,18,19] and *Salmonella Typhimurium* [20]. Therefore, standardizing biofilms in terms of cell density appears as a prerequisite in order to undertake a relevant analysis of a CAP treatment efficacy and to investigate the role of the extracellular matrix and/or the cellular compartment on biofilm resistance.

We here selected two bacterial strains: (i) *Pseudomonas aeruginosa* ATCC 15442, a Gram-negative bacterium that is recommended as a standard model for testing antimicrobial preservatives under national regulatory standards NF EN1040 (AFNOR 2006) and ASTM standards, and (ii) *Leuconostoc citreum* NRRL B-1299, a Gram-positive lactic acid bacterium which has the capacity to form thick biofilms due to the production of high-molecular weight dextrans in the presence of sucrose [21,22].

In this study, the main objectives were thus to: (i) Develop a method to standardize the production of mono-species bacterial biofilms formed by *Pseudomonas aeruginosa* and *Leuconostoc citreum* (with glucan-rich matrix or not); and (ii) investigate the influence of the extracellular matrix on the biofilm eradication potential of a plasma corona device operating in ambient air. Bacterial biofilms were standardized in terms of cell load and were characterized using dry weight, scanning electron microscopy, and ATR-FTIR spectroscopy. The bactericidal effect of the CAP treatment was then evaluated by the conventional plate count method and correlated to biofilm properties. Overall results are expected to shed light on the potential implication of extracellular matrix compounds within biofilms in response to CAP treatments.

## 2. Materials and Methods

### 2.1. Bacterial Strains and Cultivation Method

The two bacterial strains used in this study were *Leuconostoc citreum* NRRL B-1299 (ATCC 11449) and *Pseudomonas aeruginosa* ATCC 15442. This latter strain has a broad spectrum of resistance to various commercial germicides, is a non-mucoid strain, is neither invasive nor cytotoxic, and does not produce pyocyanin [23]. Stock cultures were maintained at −80 °C in glycerol (20% *v/v*) for both strains. *L. citreum* was routinely cultivated at 30 °C in MRS (de Man Rogosa and Sharpe) medium, and the *P. aeruginosa* strain was cultivated at 37 °C in the LB (Luria Bertani) medium. Control of the cell concentration was achieved by $OD_{600\mathrm{nm}}$ measurements, considering that an $OD_{600\mathrm{nm}}$ equal to 1.0 corresponded to $1.10^{8}$ CFU/mL for *L. citreum* and $3.10^{7}$ CFU/mL for *P. aeruginosa*, respectively.

### 2.2. Biofilm Formation

Biofilms were grown on sterile hydrophilic membranes (Pall GN-6 Metricel${}^{®}$ sterile membranes, diameter 47 mm, pore size 0.45 μm, with grids) as previously described by Marchal et al. [15] with slight modifications. Briefly, 20 mL of a calibrated suspension (obtained by dilution in fresh medium from an overnight suspension) were filtered through mixed cellulose ester membranes in order to homogeneously deposit the cells over the membrane surface. The membrane was then cut under sterile conditions in four equal squares of 1.5 cm${}^{2}$, and each individual coupon was immediately placed in the center of a Petri dish (55 mm diameter) containing either MRS solid media with or without sucrose (40 g/L) for *L. citreum*, and LB solid media for *P. aeruginosa*. This process gave calibrated surface-spread bacteria (SSB) samples, which will be used directly for CAP treatments or for biofilm growth. For biofilm growth, plates were incubated during 24 h at 30 °C for *L. citreum* or 37 °C for *P. aeruginosa*. The *L. citreum* biofilms grown on sucrose-containing medium will be called hereafter “glucan-rich biofilms”. The total mass of biofilms and dry weight measurements were undertaken on 24-h biofilms grown on whole 47-mm membranes, with a moisture analyzer (Sartorius MA30) and the wet and dry mass of biofilms were corrected with the mass of the membrane.

### 2.3. Plasma Source and Exposure

The used CAP system was a direct current (DC) homemade corona system as previously described in several studies [15,24,25]. The CAP was composed of an anodic tungsten needle (20 -μm radius) inside a cylindrical brass cathode (20-mm inner diameter) linked to the ground. The plasma jet was supplied by a high-voltage generator through a resistor of 25 MΩ to avoid arcing. The voltage was adjusted at 14.7 kV. In this configuration, the plasma jet was generated directly in the ambient air at atmospheric pressure and launched by itself without any system of gas inlet feed. This corona discharge has a natural repetitive discharge current with a frequency of about 20 kHz. The electric power injected was about 100 mW. The visible plasma effluent was approximately 15–20 mm long and 1–2 mm in diameter from the end of the cylindrical cathode.

Optical emission spectroscopy was used to estimate the radiative active species of the plasma effluent column generated by the corona discharge and launched on the *L. citreum* 24 h-biofilm. A spectrophotometer (Acton Spectra SP 2750, in the Czerny Turner configuration) with 0.75 m of focal length covering a spectral range lying between 200 nm and 900 nm was used in this study. The detecting device set at the exit of this spectrophotometer was a CCD camera (PIXIS 100, 1340 × 100 imaging array of 20 μm × 20 μm pixels). The plasma spectra were collected through an optical fibre (UV-silicon LG-455-020-3) placed at the spectrometer entrance slit (being 50 μm wide) and connected, on the plasma side, to an optical system (composed by 2 magnification lens) aimed to target a small plasma volume (1 mm${}^{3}$). Spectra collections were performed using a 2400 grooves/mm grating in the UV range and a 1800 grooves/mm grating in the visible range. An optical long-pass filter was used for the visible range in order to prevent second order effects caused by the grating second order diffracted beam.

Preliminary experiments were performed to evaluate the potential bacterial growth inhibition area by the plasma jet. Hence, 100 μL of an overnight *L. citreum* suspension were spread over MRS agar plates, left to dry for 15 min, and exposed for 20 min to the plasma treatment. The area of bacterial growth inhibition was measured after incubation for 24 h at 30 °C. Secondly, to examine the thermal or dehydration effect of the plasma, the temperature was measured along the axis of the plasma jet using a thermocouple, and the mass of agar plates before and after 30 min of exposure was evaluated in triplicate, respectively.

For treatment of SSB and biofilm samples, plasma was applied directly in the center of each coupon, and the agar plate was not moved during treatment. The distance between the target sample and the end of the cylindrical cathode of the plasma jet was set at 20 mm. Three coupons provided from the same membrane were treated for 5, 10, and 20 min, respectively, and the fourth coupon was left untreated (as a control sample) to estimate the initial bacterial concentration on the membrane.

### 2.4. ATR-FTIR Spectroscopy

For FTIR analysis, biofilm samples (before and after plasma exposure) were dehydrated by using a moisture analyzer (Sartorius MA30). The coupons were heated at 45 °C until reaching constant weight. The spectra of bacterial cells without matrix were obtained by analyzing dehydrated pellets of overnight microbial cultures. The extracellular glucan polymer fraction from *L. citreum* NRRL B-1299 was produced by enzymatic reaction from sucrose and purified as previously described by Bounaix et al. [21]. The FTIR spectra were recorded between 4000 and 800 cm${}^{-1}$ on a Spectrum 65 spectrometer equipped with the Spectrum software (Perkin Elmer). Each spectrum was an average of 32 scans with a resolution of 4 cm${}^{-1}$. For all spectra, baselines were corrected. Spectra were normalized on the 1280 cm${}^{-1}$ peak, which corresponds to a distinctive peak from the membrane, and with low abundance for bacterial cells and biofilm spectra. Band assignments were made according to the literature [26,27]. Moreover, in order to compare the different spectra, a ratio was calculated based on the responses obtained at 1540 cm${}^{-1}$ (amide II) and 1070 cm${}^{-1}$ (polysaccharides), which will be called hereafter AmII/PS. The amide II band was preferred to the amide I band to represent the protein fraction of the samples due to the absence of response of the membrane at this wave number. Finally, the overall similarity (p<0.05) between spectra from glucan-rich and dextrans was directly provided by the data processing software.

### 2.5. Scanning Electron Microscopy

Scanning electron microscopy (SEM) analysis was applied on bacterial biofilms, which were produced as described previously. Coupons were removed from agar plates and were desiccated under vacuum for 15 min to 1 h, depending on the samples. No fixation step or staining was performed, as previously proposed by Lackmann et al. [28]. Samples were then metallized with platinum to allow image acquisition. Analysis was performed with a MEB Quanta 250 FEG FEI at the microscopy platform, Centre de Microscopie Électronique Appliquée à la Biologie (CMEAB) in Toulouse.

### 2.6. Bacterial Viability

Membrane coupons of untreated control and plasma-exposed samples were placed into 1 mL of saline sterile solution (NaCl 9 g/L) and vigorously mixed by vortexing 1 min, which disrupted biofilms and released bacterial cells. Serial ten-fold dilutions were then achieved on the obtained bacterial suspensions, and CFU counts were determined in duplicate on appropriate culture media. The efficacy of this initial recovery step was checked by counting the bacterial cells remaining on the membrane coupon: Coupons were once again placed into 1 mL of sterile physiological solution and treated as described above. These experiments showed that more than 95% of cells could be recovered from the coupon by applying the one-step procedure.

Cultivable cell reduction values were calculated by subtracting the log CFU cm${}^{-2}$ of treated coupons from the log CFU cm${}^{-2}$ of their own control coupon i.e., provided from the same membrane sample.

### 2.7. Statistical Analysis

All experiments were conducted in triplicate, with independent initial bacterial culture, and results were expressed as mean ± SEM. A one-way ANOVA was conducted and the Newman–Keuls multiple comparison test was used to determine the level of significance against the untreated control value (p<0.05).

## 3. Results

### 3.1. Standardization and Characterization of the Bacterial Model Biofilms

**Figure 1:**
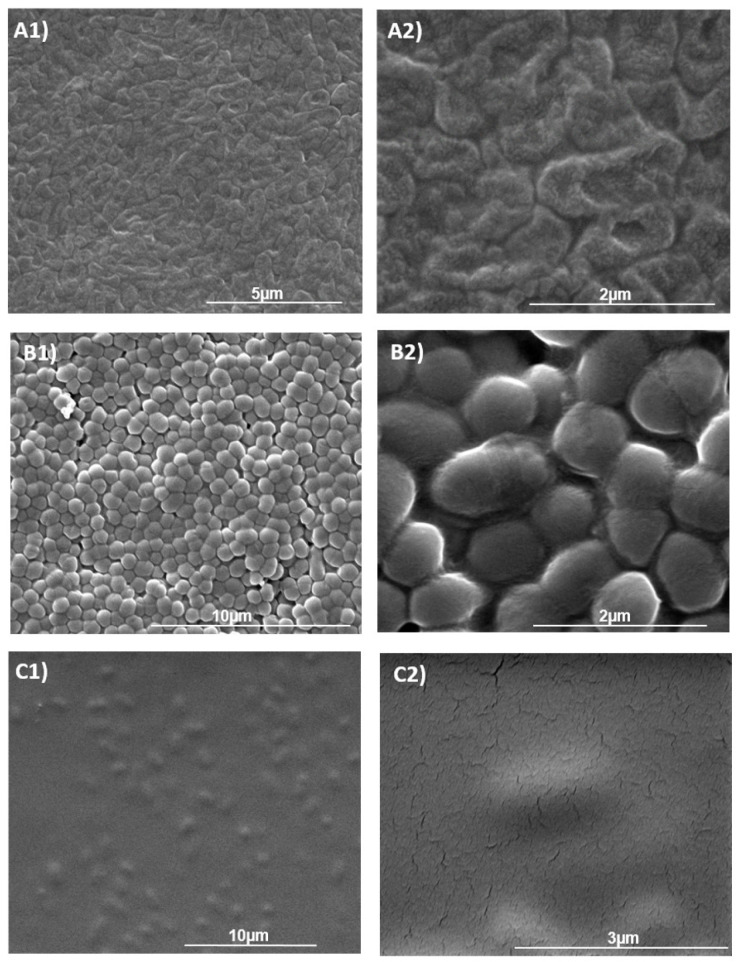
SEM observations of the 24-h biofilms formed on mixed cellulose ester membrane coupon. (**A**) *P. aeruginosa* biofilm on LB medium; (**B**) *L. citreum* biofilm on MRS medium, (**C**) *L. citreum* biofilm on sucrose (40 g L${}^{-1}$) containing MRS. (**A1**–**C1**) at low magnification and (**A2**–**C2**) at high magnification.

The methodology applied to produce the biofilms on the membrane led to significantly comparable cell loads with concentrations reaching 8 log CFU cm${}^{-2}$ for both *L. citreum* and *P. aeruginosa* biofilms (Table 1). Although similar cell densities were obtained for both *L. citreum* biofilms, whether produced with sucrose or not, dry weight, on the other hand, was 4-fold higher for biofilms cultivated on sucrose supplemented medium, demonstrating the production of glucan polymers (Table 1). All the biofilms were well-hydrated with moisture contents of 82% and 92% for *L. citreum* (glucan-rich or not, respectively) and 90% for *P. aeruginosa*. SEM also allows one to visualize the homogeneous distribution of *L. citreum* and *P. aeruginosa* cells on the membrane coupons (Figure 1). Images showed that all biofilms obtained after 24 h of culture were composed of densely arranged cells, covering the whole surface of the membrane. However, biofilms were physically different: A layer of smooth distinct cells for *L. citreum* biofilm grown on MRS media, cells embedded in a thick slimy matrix due to the overproduction of glucans for *L. citreum* biofilm grown on sucrose supplemented media, and a rough surface for *P. aeruginosa* biofilm.

**Figure 2:**
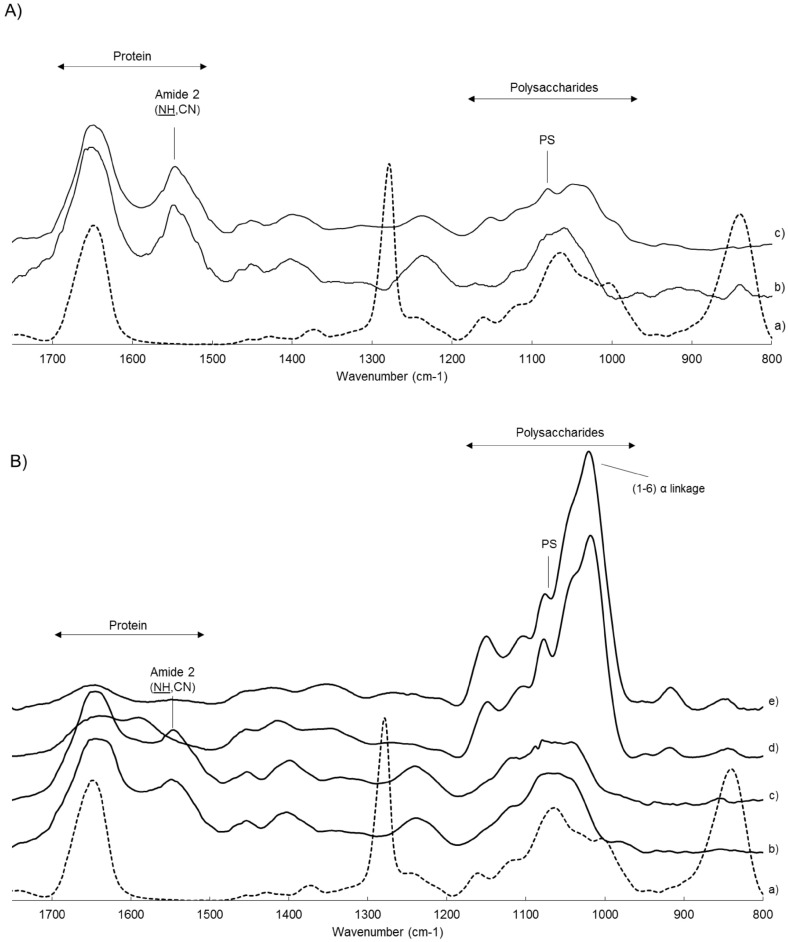
ATR-FTIR spectra of 24-h biofilms compared to planktonic cells and membrane coupon. (**A**) *P. aeruginosa* spectra: (a) Uninoculated membrane, (b) cells of *P. aeruginosa*, and (c) 24-h biofilm. (**B**) *L. citreum* spectra: (a) Uninoculated membrane, (b) cells, (c) 24-h biofilm, (d) glucan-rich 24-h biofilm, and (e) purified glucan polymer. Each spectrum undergoes a baseline correction and for better visibility, each absorbance was expressed in total absorbance percentage and spectra have been vertically shifted.

ATR-FTIR spectroscopy confirmed that the membrane support is well covered by the biofilms (Figure 2). Indeed, distinctive peaks of the mixed cellulose ester membrane that could be observed at 1280 cm${}^{-1}$ and 840 cm${}^{-1}$ were no longer visible after a 24-h biofilm development (Figure 2). The value of the amide II/polysaccharide (AmII/PS) ratio measured at 1540 cm${}^{-1}$ and 1070 cm${}^{-1}$ peaks (Table 2) revealed that proteins are relatively more present in *P. aeruginosa* cells (average ratio 1.28) than in *L. citreum* cells (average ratio 0.88). The corresponding 24-h biofilms presented a slight increase in protein content compared to the cells alone. *P. aeruginosa* biofilm maintained higher protein content (average ratio 1.39) whereas *L. citreum* biofilm showed a more equilibrated pattern between proteins and polysaccharides (average ratio 1.14). Obviously, a large proportion of polysaccharides, shown as intense peaks ranging from 1200 to 1000 cm${}^{-1}$ was detected on *L. citreum* biofilms grown on a sucrose-containing medium, with an AmII/PS ratio of only 0.16. FTIR measurements on purified glucan fractions produced by planktonic *L. citreum* cells grown on sucrose showed a similar spectrum (correlation coefficient of 95%), confirming the abundance of glucans in the ECM.

### 3.2. Characteristics of Corona Plasma Jet

**Figure 3:**
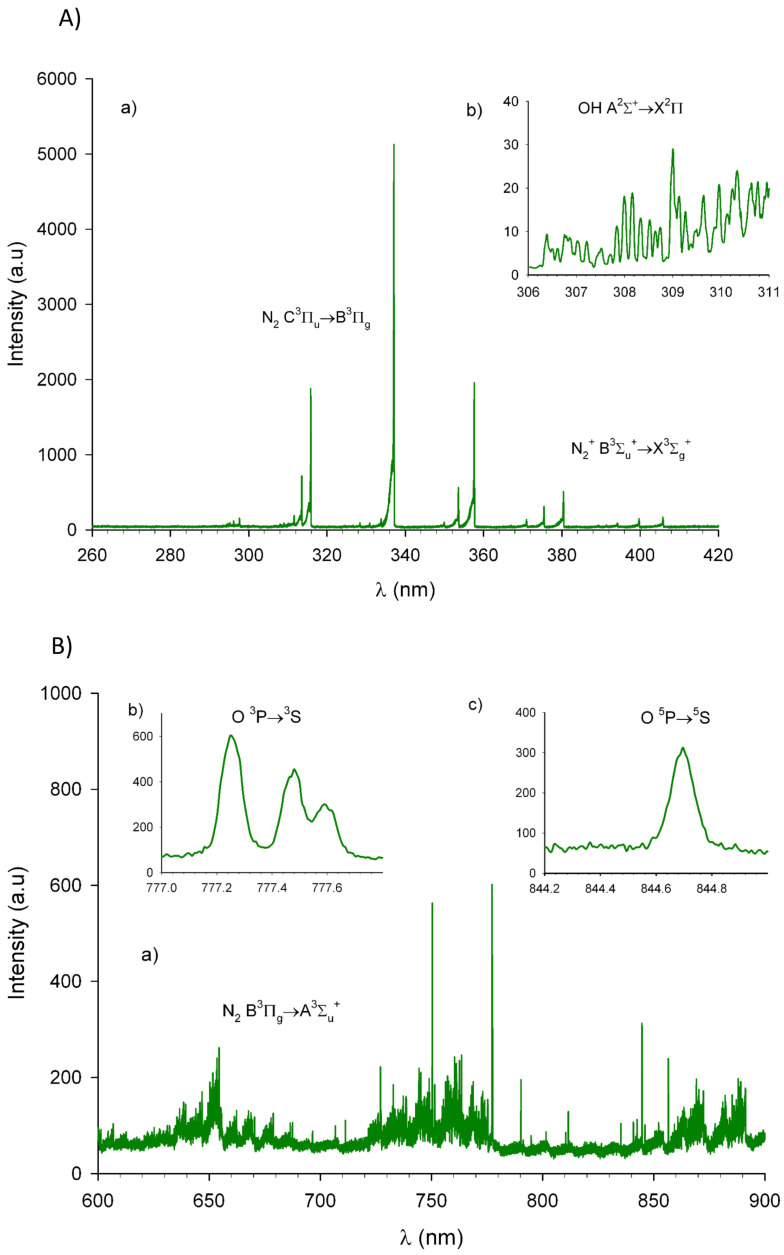
Emission spectra of corona plasma around the needle of the plasma device during *L. citreum* biofilm treatment. (**A**) Emission spectra in the UV range. No emission was detected for a wavelength lower than 250 nm. (a) Observation of the second positive nitrogen system from 290 nm to 440 nm and the first negative nitrogen system around 392 nm, (b) OH(A-X) emission from 306 nm to 312 nm recorded with an exposure time 300-fold higher. (**B**) Emission spectra in the visible range. (a) Observation of the first positive nitrogen system from 650 nm to 750 nm, (b) emission of atomic oxygen at 777 nm, and (c) at 844 nm.

Emission spectra were recorded during biofilm treatments (Figure 3). Overall, results revealed the presence of excited molecular species like nitrogen $N_{2}$, molecular ion of nitrogen $N_{2}^{+}$, and free radicals of OH and O in the vicinity of the bacterial biofilm during the plasma treatment. In particular, emissions of atomic oxygen could be observed at 777 and 844 nm. No emissions were detected at a wavelength below 250 nm, indicating that no UV-C photons had been produced in our plasma effluent. Ozone concentration, produced by the reaction of atomic oxygen with the molecular oxygen of the surrounding environment, was estimated 60–80 ppm nearby the sample (data not shown). Measured temperature at the surface of the biofilm did not exceed 27 °C, thereby precluding the heat effect from the plasma. Inactivation of bacteria can be here also excluded from a dehydration effect. Indeed, the loss of water content was estimated at less than 3% when mass of agar plates was compared before and after 30 min of treatment. This can be attributed to the ionic wind jet produced by the plasma regarding DC corona discharge configuration.

### 3.3. Cold Plasma Jet Treatment of Surface-Spread Cells

**Figure 4:**
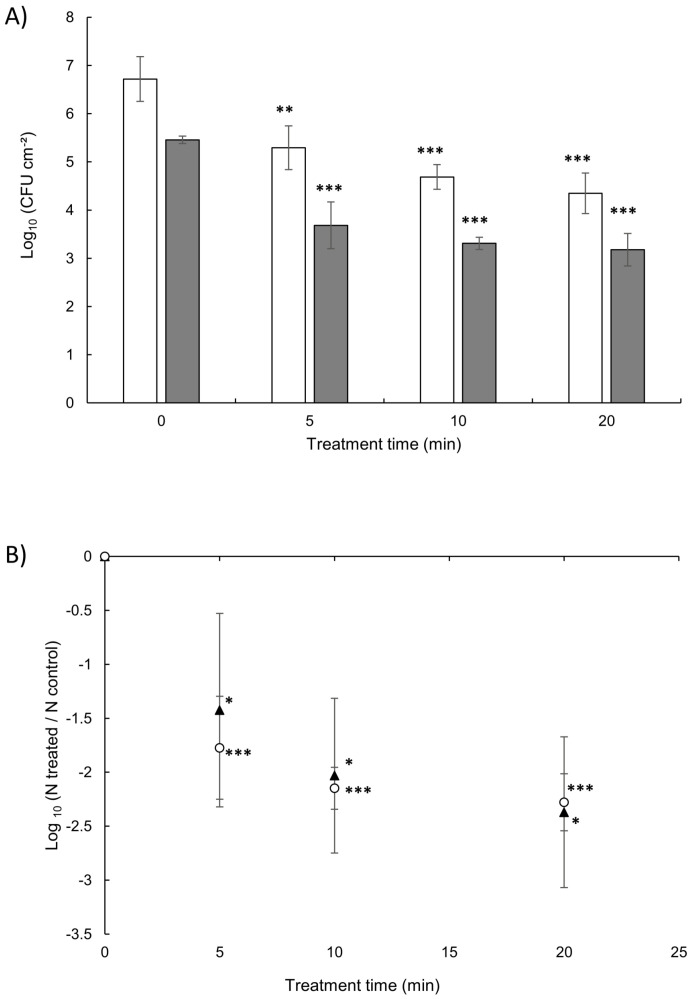
Survival curves for surface-spread bacteria after plasma treatment. (**A**) Bacterial population in CFU cm${}^{-2}$ before and after plasma exposure from 5, 10, and 20 min for *P. aeruginosa* (grey bar) and *L. citreum* (open bar); (**B**) logarithmic reduction of the bacterial population for *P. aeruginosa* (*▲*) and *L. citreum* (∘). Data represent means ±sd from three independent experiments (*, p<0.05, **, p<0.01 and ***, p<0.001 against the negative control as measured by the Newman–Keuls test).

Preliminary experiments were conducted with *L. citreum* to determine the surface targeted by the plasma effluent in terms of the bacterial growth inhibition area. Even if the diameter of air plasma jet is about 2 mm, the plasma inhibited the growth of bacterial cells over a circular surface of higher diameter (28±1 mm, n=3), probably due to the spreading of the active species generated by the plasma jet into the agar medium. As this inhibition area was really larger than the square coupon surface (150 mm${}^{2}$), it could be suggested that all bacteria deposited or grown on the coupons can be affected by the treatment. For both strains, the eradication efficacy of surface-spread bacteria (SSB) increased with the time of treatment, with a maximal reduction reached after 20 min (Figure 4). For *P. aeruginosa*, a significant 1.8 log bacterial reduction could be observed from a 5-min plasma exposure, to a maximum reduction of 2.3 log after 20 min. A similar response was observed for *L. citreum*: Approximately 1.4 log after 5 min of treatment to a maximum of 2.4 log reduction after 20 min. Extended treatment time was investigated up to 40 min on both strains and results did not reveal an increase in efficacy (data not shown).

### 3.4. Viability of Biofilm Cells after Plasma Exposure

**Figure 5:**
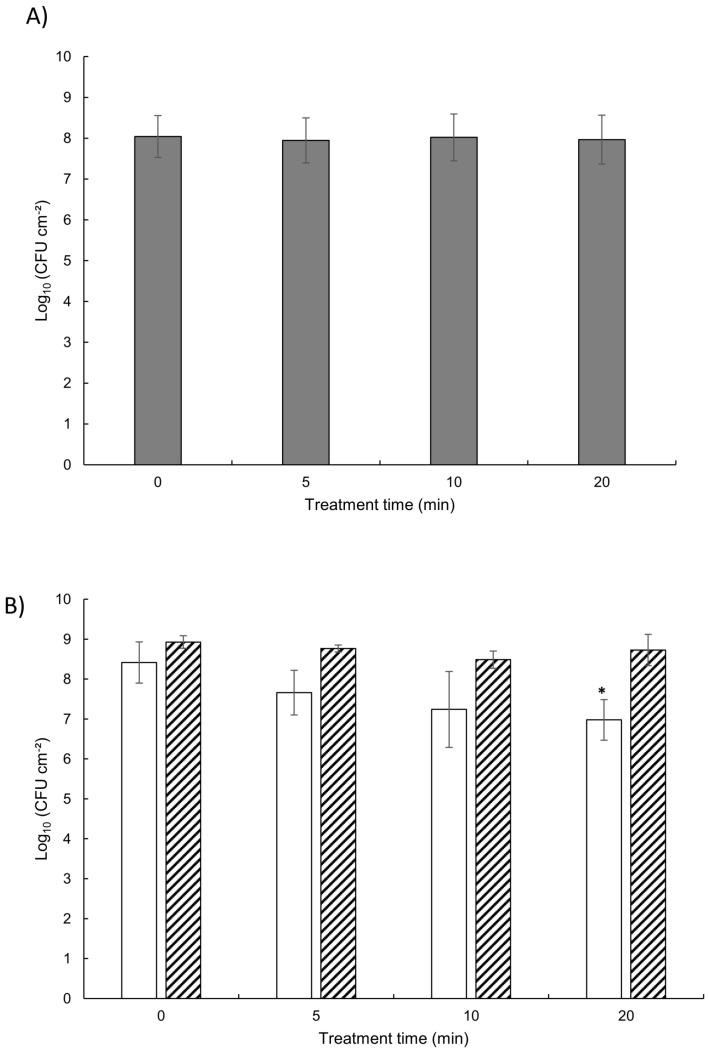
Survival of bacteria in 24-h biofilms after plasma exposure. CFU cm${}^{-2}$ count on membrane coupon before and after plasma exposure for 5, 10, and 20 min for (**A**) *P. aeruginosa* and (**B**) *L. citreum* grown without (open bar) or with (hatched bar) sucrose. Data represent means ±sd from three independent experiments (*: p<0.05 against the negative control as measured by the Newman–Keuls test).

When 24-h *P. aeruginosa* biofilms were treated in the same conditions as above, no bactericidal effect could be observed even after 20 min of treatment (Figure 5). Similar results were obtained when *L. citreum* biofilms were produced in the presence of sucrose, i.e., with a high production of extracellular glucan polymers. Conversely, CAP exposure was significantly effective to reduce 24-h *L. citreum* biofilms with a similar cell density than the previously described biofilms, by a 1.5 log reduction after 20 min of treatment. SEM images of *L. citreum* and *P. aeruginosa* biofilms after plasma exposure did not reveal noticeable differences in cell size, shape, and surface structure of treated cells versus untreated controls (data not shown). This is relevant with the observations of Soler-Arango et al. [29] who imaged *P. aeruginosa* PAO1 biofilms treated with plasma for 0, 3, and 30 min. These authors did not observe significant damage either to the matrix or to the cell morphology for a 3-min treatment, even if a disorganization of the biofilm with distorted and broken cells and matrix disintegration appeared for the highest dose. The comparison between normalized ATR-FTIR spectra of *L. citreum* and *P. aeruginosa* biofilms before and after a 20-min plasma jet treatment (Figure S1) revealed that only *L. citreum* biofilm (without sucrose addition) showed a significantly different profile. However, we could observe that the AmII/PS ratio decreased by approximately 30 to 40% for *P. aeruginosa* and *L. citreum* biofilms, respectively (Table 2), indicating in both cases a relative loss of protein residues. These observations are relevant with the results of Soler-Arango et al. [29] who provided evidence that the biofilm exposure to plasma produced significant modifications in the biomass content and denaturation or aggregation of proteins at the highest exposure time.

## 4. Discussion

In this study, we aimed at highlighting the potential implication of the extracellular matrix in the response of 24-h biofilms to CAP treatment, while also controlling cell density to about a similar 8 log CFU/cm${}^{2}$ for all samples. *P. aeruginosa* and *L. citreum* strains were selected to target biofilm-forming bacteria with different cell wall structures, respectively Gram-negative and Gram-positive, and also because these two bacterial species present contrasted extracellular matrices when growing into biofilms, as described in detail below. To date, there is still no consensus on the plasma resistant property of Gram-positive versus Gram-negative bacteria [8], and the ECM is still rarely taken into account as a parameter potentially affecting CAP efficacy.

As judged by MEB observations and ATR-FTIR results, the methodology applied in this study clearly provided uniform and homogeneous biofilms covering the whole coupon surface, with similar cell loads but different extracellular matrices. Deposition of cells obtained by calibrated suspensions through filtration on the membrane coupon, rather than by simple surface deposits, certainly contributed to the formation of a homogeneous biofilm, as previously reported by Bayliss et al. [30]. Interest in the initial microbial load of biofilm is, to date, rarely considered, especially that bacterial density can vary from 3 to 9 log cm${}^{-2}$ depending on the study, and sometimes vary even within the same study, with values more often below 6 log [13,31,32,33]. In addition, the contribution of cell density could be masked when the expression of plasma efficacy is only reported by a single logarithmic reduction.

The (untreated) biofilm characterization by a quantitative approach, using the dry weight of 24-h biofilms, clearly showed the importance of the ECM: The dry weight values of the *L. citreum* glucan-rich biofilms were 2-fold higher than the *P. aeruginosa* biofilms, and 4-fold higher than the *L. citreum* biofilms grown on the MRS medium alone.

In addition, ATR-FTIR spectroscopy was selected for its rapid and broad in situ analysis of the biofilms by providing qualitative information on the nature of the components of the matrix, and also relative quantitative information of some type of compounds [26,34]. Despite inherent complexity of ATR-FTIR bacterial spectra, this technique has been successfully employed to monitor the early stages of biofilm formation [26,34] and also once recently employed to investigate biofilm eradication by plasma technology using a submerged dielectric barrier discharge plasma [27]. In our study, the calculated AmII/PS ratio revealed that the biofilm produced by *P. aeruginosa* ATCC 15442 was rich in proteins. This is consistent with previous reports that showed the presence of abundant proteins, especially amyloids fibrils, along with extracellular DNA and polysaccharides within the biofilm matrix of this strain in a microplate assay [35,36], and as usually described for others non-mucoid *P. aeruginosa* strains [37,38]. On the contrary, *L. citreum* biofilms have been poorly studied so far [39,40], however this present study showed that *L. citreum* NRRL B-1299 produced biofilms with a lower relative protein content. Furthermore, the extracellular glucane-saccharases produced by this strain allow to control the production of abundant glucan exopolymers of the dextran type [22,41]. This is achieved in a very easy and simple way, since adding sucrose into the culture medium provides the only substrate required by these extracellular enzymes. It was also shown here that purified glucan polymer fraction from planktonic culture and glucan-rich *L. citreum* 24-h biofilm both presented similar spectra with a notable intense peak at 1020 cm${}^{-1}$, characteristic of α−(1,6) glucosidic linkages which are abundant in dextran polymers [42].

The main concern of our work was to study the implication of the ECM in the biofilm response to CAP treatment. Treatment of surface-spread cells of *P. aeruginosa* and *L. citreum* (without any incubation period) revealed a significant antibacterial time-dependent effect of the CAP treatment. Indeed the cultivability of the bacteria was reduced significantly from 5 min of treatment to a maximal effect reaching less than 1% survival cells after 20 min of exposure. These results confirmed a previous report on evaluating the effect of a similar plasma device with another LAB strain [15]. Unlike some other reports [31,32], we did not observe a higher sensitivity of the Gram-negative *P. aeruginosa* strain to plasma exposure, compared to the Gram-positive *L. citreum*. Actually, it is often argued that Gram-negative bacteria are more CAP sensitive due to the inherent thin layer of the peptidoglycan which composes its cell wall [31,32]. However, other factors such as the overall thickness and the other compounds of the cell envelope can also be taken into account [43].

When treating 24-h biofilms with an initial load of approximately 8 log bacteria CFU cm${}^{-2}$, a significant time-dependent cultivability reduction was only observed for *L. citreum* biofilms but without any bactericidal efficacy on *P. aeruginosa* and glucan-rich *L. citreum* biofilms. Accurate direct comparison with the results obtained from the surface-spread condition is rather difficult since the initial microbial load was higher (by a factor 40 to 200) due to the used protocol, which was developed to obtain constant cell density biofilms after 24 h. Therefore, plasma inefficacy within biofilms in respect to SSB could be here attributed to both a higher microbial load and the presence of matrix compounds. However, as initial cell loads for the three biofilms were not significantly different, and also because of their specific biomasses, results obtained in this study thus highlighted the contribution of the ECM in response to CAP treatments. In addition, the data of plasma-induced bacterial reduction for *L. citreum* biofilms grown with or without the addition of sucrose were statistically different, indicating that production of glucans clearly impaired plasma efficacy. Data showed that the cultivability reduction could be explained quite well ($r^{2}=0.88$) by taking into account simultaneously the treatment time as well as initial matrix properties (biofilm AmII/PS ratio and total dry weight).

It is generally accepted that the antimicrobial properties of cold plasma are predominantly attributed to the production of reactive oxygen and nitrogen species (RNOS) and in some cases to UV radiations [7,8,9]. These were not present in the plasma jet under the experimental conditions of this study, thus excluding their action. On the other hand, optical emission spectra showed that the plasma jet produced reactive oxygen species (ROS) such as O and OH and reactive nitrogen species (RNS) such as $N_{2}^{*}$ and $N_{2}^{+}$. These reactive species generated during biofilm contact were similar to what has been previously detected when applying this specific plasma device simply in the air [15]. Only OH emissions (in the spectral region from 306 to 312 nm) were different, due to the ambient humidity caused by the presence of agar media under the coupon. These species generated in the plasma, might also react with the liquid surrounding the bacteria leading to form antibacterial by-products such as peroxides and superoxides [4,6].

The exact mechanisms underlying bactericidal inactivation by cold plasma are still unclear and it is assumed that multiple actions due to the diversity of active agents are playing a role in the permeabilization of cell walls and/or membranes, and in protein damage and enzyme inactivation [7,8,9]. RNOS are known to exhibit strong oxidative properties, and are able to degrade biomacromolecules, including proteins, lipids, nucleic acids, as well as polysaccharides [44], present both as cellular and matrix components of biofilms. The role of excited $N_{2}^{*}$ species is expected to be less significant in the bacteria-inactivation process compared to ROS species [45]. In agreement with these assumptions, reduced cell viability after CAP treatment revealed to be time-dependant for surface-spread bacteria as well as for biofilms exposing low extracellular compound contents. Moreover, despite the absence of bactericidal effect on biofilms presenting thick matrices, ATR-FTIR measurements revealed a 30% reduction in the amount of protein residues (as evaluated by amide II band) in the *P. aeruginosa* biofilm after 20 min of plasma exposure. This is in agreement with Khan et al. [27] who showed that the protein peak region was reduced by 10 to 20% (also based on AmII/PS ratio) after exposing three different biofilms to a DBD plasma reactor which eradicated 90% of the bacterial population. In addition, reports on in vitro plasma exposures of purified proteins showed diverse structural alterations [6,8].

One frequent argument to explain why plasma efficacy upon biofilms is reduced, is the presence of the extracellular matrix, who acts as a physical barrier to antimicrobials, thereby preventing the penetration of plasma active species [4]. However, ROS species are expected to easily penetrate the biofilms and impact the bacteria deep inside them. For instance, it was reported that ROS generated by plasma were able to penetrate right through to the bottom layer of a 25.5μm thick *Enterococcus faecalis* biofilm when using a plasma jet, operating in open air [46]. Components of the matrix can also act as a chemical barrier protecting the cells by possible scavenging of biocidal compounds, as previously mentioned [33,47]. Accordingly, Yan et al. [48] recently reported that extracellular glucan produced by *Leuconostoc mesenteroides* could improve the survival of cells during chronic oxidative stress. Other polysaccharides, such as alginate, a heteropolysaccharide excreted in mucoid *P. aeruginosa* matrix, are also likely to be involved in scavenging hydroxyl radicals (OH) and $H_{2}O_{2}$ [49]. As evidence, Gilmore et al. [50] reported that the addition of 1.25% alginate to planktonic cells of *P. aeruginosa* PA01 increased the survival from 52% to 91% after a DBD plasma jet exposure. These results on planktonic *P. aeruginosa* PAO1 cells were recently confirmed: A protective effect was observed, at lower exposure times to an atmospheric pressure nonthermal plasma (APNTP), after the addition of exogenous alginate or DNA, with an additive protective effect with alginate and DNA together [51]. These authors also reported that the endogenous production of alginate by a *P. aeruginosa* mutant strain overexpressing alginate to form biofilms that had a protective effect against the antimicrobial action of the plasma treatment [51].

Overall results obtained in this study indicate that the ECM in thick biofilms, such as *P. aeruginosa* and glucan-rich *L. citreum* biofilms has most probably a role in the resistance to plasma treatment. This is in agreement with previous studies that have shown that (i) clinical isolates of *Burkholderia cepacia* producing the highest amount of biomass were more tolerant to the biocide effect of CAP [52], and (ii) that *Acinetobacter baumannii* biofilm biomass mediates tolerance to cold plasma [53].

In conclusion, this study allowed to produce similar biofilms in terms of cell densities but are different in terms of matrix composition, and thus to evaluate the contribution of the ECM in the response to CAP treatments. Notably, these in vitro 24 h-old mono-species biofilms did not represent complex natural biofilms, but were used as standardized tools that are able to produce highly reproducible biofilms for accurate comparative laboratory scale studies. In these conditions, the experiments showed that the characteristics of extracellular matrix in which cells are embedded drastically affect CAP sensitivity. The ECM of biofilms is complex and varies depending on the type of bacteria (species and strain) within the biofilm and on the growth conditions. The ECM should be thus considered, along with the microbial load, when studying the effectiveness of CAP technology. Further studies are now required to better understand the interactions of plasma reactive species within the organic matrix of biofilms.

## Figures and Tables

**Table 1:** Biofilm characteristics regarding cell density and biomass. Bacterial population was recovered from untreated 24-h biofilms formed on mixed cellulose esters membrane coupon and expressed as log CFU cm${}^{-2}$. *n*: replicate number.

	*P. aeruginosa*	*L. citreum*	
		without Sucrose	with Sucrose
Cells density (log CFU cm${}^{-2}$ )	8.07±0.46 (n=6)	8.36±0.45 (n=8)	8.8±0.26 (n=6)
Wet mass (mg)	314±21 (n=3)	255±21 (n=3)	466±23 (n=3)
Dry mass (mg)	35.8±5.9 (n=3)	20.3±2.5 (n=3)	82±9.9 (n=3)

**Table 2:** Intensity of the Amide II/Polysaccharide ratio for planktonic cells and biofilms. Ratio was calculated from ATR-FTIR spectra for wave numbers 1540 cm${}^{-1}$ (Amide II) and 1070 cm${}^{-1}$ (polysaccharides) after normalization of the spectra on a distinctive peak of the membrane (1280 cm${}^{-1}$).

	*P. aeruginosa*	*L. citreum*	
		without Sucrose	with Sucrose
Cells	1.28	0.88	
24 h biofilm-untreated	1.39±0.03	1.14±0.04	0.16±0.01
24 h biofilm-treated 20 min	0.95±0.20	0.63±0.20	0.13±0.04

## Data Availability

Not applicable.

## Supplementary Materials

The following are available online at https://www.mdpi.com/article/10.3390/life11070694/s1, Figure S1: Comparison of ATR-FTIR spectra from untreated and cold plasma treated 24 h-biofilm samples (A) *P. aeruginosa* biofilm, (B) *L. citreum biofilm*, and (C) glucan-rich *L. citreum* biofilm. In each figure, uninoculated membrane ( ··· ), untreated sample ( —– ), and treated for 20 min (——). Ranging from 1800 to 800 cm${}^{-1}$, ATR-FTIR spectra were normalized on the peak of the membrane at 1280 cm${}^{-1}$.
